# Advancements in Bio-Nanotechnology: Green Synthesis and Emerging Applications of Bio-Nanoparticles

**DOI:** 10.3390/nano15070528

**Published:** 2025-03-31

**Authors:** M. D. K. M. Gunasena, G. D. C. P. Galpaya, C. J. Abeygunawardena, D. K. A. Induranga, H. V. V. Priyadarshana, S. S. Millavithanachchi, P. K. G. S. S. Bandara, K. R. Koswattage

**Affiliations:** 1 Department of Biosystems Technology, Faculty of Technology, Sabaragamuwa University of Sri Lanka, Belihuloya 70140, Sri Lanka; kasundi@tech.sab.ac.lk (M.D.K.M.G.); sahani@tech.sab.ac.lk (S.S.M.); sarath@agri.sab.ac.lk (P.K.G.S.S.B.); 2 Center for Nanodevice Fabrication and Characterization, Faculty of Technology, Sabaragamuwa University of Sri Lanka, Belihuloya 70140, Sri Lanka; chanakagalpaya@gmail.com (G.D.C.P.G.); ashaninduranga@tech.sab.ac.lk (D.K.A.I.); vimukkthi@tech.sab.ac.lk (H.V.V.P.); 3 Department of Chemistry and Biochemistry, Baylor University, Waco, TX 76706, USA; charitha_abeygunawa1@baylor.edu; 4 Department of Engineering Technology, Faculty of Technology, Sabaragamuwa University of Sri Lanka, Belihuloya 70140, Sri Lanka

**Keywords:** bio-nanoparticles, green synthesis, therapeutic properties, fuel cells, energy generation, wastewater treatment, characterization

## Abstract

The field of bio-nanotechnology has seen significant advancements in recent years, particularly in the synthesis and application of bio-nanoparticles (BNPs). This review focuses on the green synthesis of BNPs using biological entities such as plants, bacteria, fungi, and algae. The utilization of these organisms for nanoparticle synthesis offers an eco-friendly and sustainable alternative to conventional chemical and physical methods, which often involve toxic reagents and high energy consumption. Phytochemicals present in plant extracts, unique metabolic pathways, and biomolecules in bacteria and fungi, and the rich biochemical composition of algae facilitate the production of nanoparticles with diverse shapes and sizes. This review further explores the wide-ranging applications of BNPs in various fields like therapeutics, fuel cells, energy generation, and wastewater treatment. In therapeutics, BNPs have shown efficacy in antimicrobial, anti-inflammatory, antioxidant, and anticancer activities. In the energy sector, BNPs are being integrated into fuel cells and other energy generation systems like bio-diesel to improve efficiency and sustainability. Their catalytic properties and large surface area enhance the performance of these devices. Wastewater treatment is another critical area where BNPs are employed for the removal of heavy metals, organic pollutants, and microbial contaminants, offering a cost-effective and environmentally friendly solution to water purification. This comprehensive review highlights the potential of bio-nanoparticles synthesized through green methods. It highlights the need for further research to optimize synthesis processes, understand mechanisms of action, and expand the scope of their applications. BNPs can be utilized to address advantages and some of the pressing challenges in medicine, energy, and environmental sustainability, paving the way for innovative and sustainable technological advancements in future prospects.

## 1. Introduction

**Figure 1:**
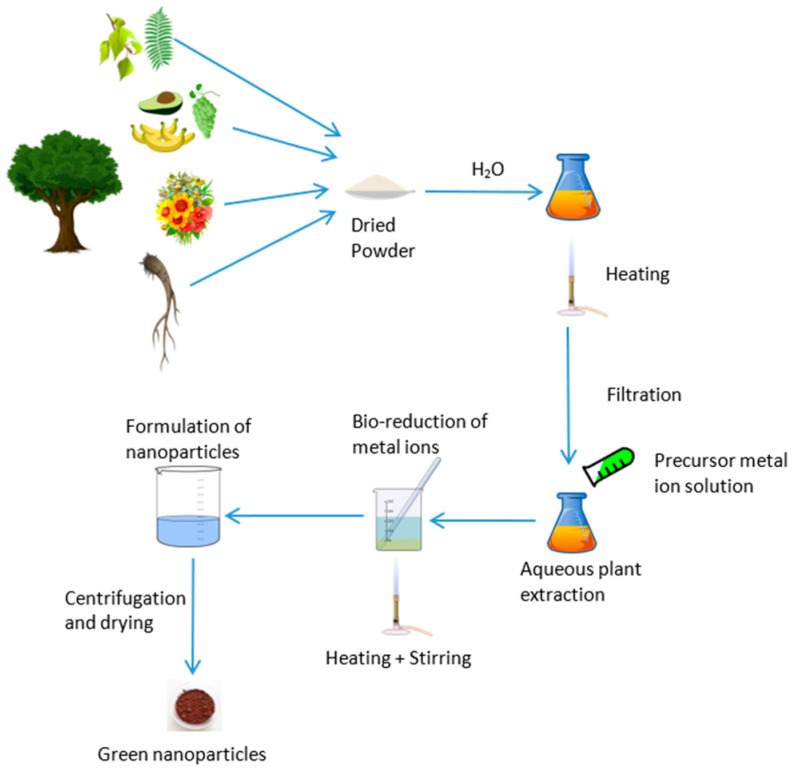
Green synthesis of bio-nanoparticles from plants.

Nanotechnology research and studies have advanced rapidly worldwide, and the applications of nanoparticles (NPs) in various fields, including biomedical applications, cell labeling, drug delivery, plant tissue culture, biomarkers, the automobile industry, and the energy sector, have become significant subjects of study in recent years, [1,2,3]. Different synthesis methods can be used for the preparation of NPs with variations in size and morphology. Chemical and physical methods are widely used, while biological methods are currently emerging as an alternative [4]. The use of chemical agents such as sodium hydroxide, sodium borohydride, potassium hydroxide, and hydrazine for reduction purposes is common in chemical methods [5,6,7,8,9], and condensation, laser ablation, laser pyrolysis, evaporation lithography, and ball milling are widely used in physical methods [10,11,12,13,14,15,16,17] for NP synthesis. Bio nanoparticle synthesizing is a sustainable solution in the nanotechnology discipline since it uses renewable and biodegradable resources (Figure 1). 

According to [18], no exact mechanism has been explained for the phytosynthesis of metallic nanoparticles. Similarly, [19] stated that identifying the precise biochemical reactions involved in the green synthesis of metallic nanoparticles remains a challenge. The general method for plant-based nanoparticle production is as follows: first, a plant and its specific part are selected and then crushed, and the plant extract is obtained. The plant extract is processed to remove any impurities. The precursor, typically a metallic solution, is then mixed with the plant extract, resulting in the production of nanoparticles. Maintaining appropriate pH, temperature, and continuous stirring (which ensures the production of uniformly sized nanoparticles) is crucial to facilitate the reaction effectively. A color change in the plant extract can be considered an indication of nanoparticle formation in some nanoparticles, such as Ag and Au, due to surface plasmon resonance (SPR) [20,21,22]. The color change observed during the reaction process serves as an indicator of nanoparticle formation. This change occurs due to SPR, where light interacts with the nanoparticles, causing them to display a different color compared to the bulk material. In addition to SPR, the quantum confinement effect also plays a role in the color variation observed during the synthesis of metallic nanoparticles [23,24].

Microorganisms produce various essential enzymes, while plants contain a range of secondary metabolites, such as phenols, terpenes, and alcohols. These enzymes and metabolites can act as reducing agents, facilitating the synthesis of nanoparticles. Additionally, plant extracts can function as stabilizers, eliminating the need for additional stabilizing agents in the solution [20,21,25]. Ref. [26] reported the presence of phytochemicals such as flavonoids, saponins, triterpenes, and steroids in *Tithonia diversifolia*. Similarly, [22] confirmed that the presence of functional groups of carbon (C) and oxygen (O) contributes to the stabilization and reduction processes involved in nanoparticle synthesis.

Plant extracts can also function as capping agents, stabilizing nanoparticles during synthesis. FTIR analysis has confirmed the involvement of various carbon (C), hydrogen (H), and oxygen (O) bonds in plant extracts, which contribute to the capping process [22,27,28]. Polyphenols, which contain multiple hydroxyl (-OH) groups attached to aromatic rings, are highly reactive in chemical reactions. For example, during the synthesis of gold nanoparticles, neighboring hydroxyl groups (typically in the ortho position) in polyphenols bind with gold ions, forming a stable five-membered chelate ring. The ortho-dihydroxyl groups (two -OH groups on adjacent carbons) are oxidized into quinones (C=O groups), while gold ions are reduced (gain electrons) to neutral gold atoms (Au^0^). This reduction occurs due to the high redox potential of gold [19,20]. Additionally, [19] reported that proteins act as stabilizing agents by providing carbonyl (-C=O) groups. These amino acid residues surround the nanoparticles, preventing aggregation and ensuring stability. FTIR analysis has provided supporting evidence for this stabilization mechanism [24].

The hydrogen radical donates its unpaired electron to silver ions (Ag^+^) in the solution, reducing them to neutral silver atoms (Ag). These silver atoms then cluster together, forming silver nanoparticles (Ag NPs). Following this reduction process, the leftover eugenol molecule, now containing a phenoxy radical on its oxygen atom, undergoes resonance stabilization. This stabilization occurs as the unpaired electron on the oxygen atom delocalizes across the benzene ring and its double bonds, making the radical more stable and less reactive. These stabilized radicals remain dissolved in the solution, aiding both nanoparticle formation and stabilization [19,29]. Ref. [30] reported that in polyphenolic compounds, neighboring hydroxyl groups form a five-membered chelate ring. Due to the extremely high oxidation-reduction potential of Au^3+^, the chelated ortho-dihydroxy groups are oxidized to quinones, while Au^3+^ is simultaneously reduced to Au. The formation of Au NPs occurs through the aggregation of nearby Au atoms, and quinones and polyphenolic compounds subsequently stabilize these nanoparticles. However, there exists several research areas for further development; for example, the efficiency of various natural resources for the green synthesis of nanomaterials has not been fully studied. Importantly, the negative impacts of those nanomaterials are also not sufficiently understood. Therefore, it is mandatory to focus on risk management throughout production, processing, preservation, and discharge [31,32]. Furthermore, the green synthesis of NPs using biological materials and their properties are summarized in Table 1.

## 2. Applications of Bio-Nanoparticles

Bio-nanomaterials offer significant advantages such as biocompatibility, biodegradability, and enhanced biological functionality, making them ideal for several applications in energy storage, environmental remediation, and medicinal applications. However, several challenges still exist, such as synthesis complexity, stability issues, and scalability constraints that need to be addressed through advanced fabrication techniques, hybrid material development, and computational modeling to enhance their performance and applicability.

### 2.1. Applications of Bio-Nanoparticles in Fuel-Cells

The fuel cell was first introduced by Sir William Grove in the 1830s. Even though the fuel cell has a long history, nowadays, many research works are being carried out that are relevant to fuel cells compared to previous decades [108,109]. The fuel cell is an effective energy converter compared to other relevant energy sources, and it only emits water and heat, making it a more environmentally friendly solution. Due to their higher energy efficiency, fuel cells are currently used in several applications in electric vehicles, alternative power sources, energy-storing methods, and space programs [110,111].

Proton exchange membrane fuel cells (PEMFs), solid-oxide fuel cells (SOFs), alkaline fuel cells (AFCs), phosphoric acid fuel cells (PAFCs), direct methanol fuel cells (DMFC), and molten carbonate fuel cells (MCFCs) can be identified as the different fuel cells types that are currently at the development. These fuel cell types are used in different applications based on their power ratings and operating temperatures. Apart from conventional fuel cells, microbial fuel cells are also being developed by scientists and can also be used as fuel cells, which is an eco-friendly solution. Microbial fuel cells can generate electricity while purifying wastewater using the metabolism power of bacteria.

Apart from the anode, cathode, and electrolyte, electro-catalysts are used in fuel cells to increase the rate of reactions in the fuel cells [112]. Most of the catalysts are noble nanoparticles such as platinum (Pt) and platinum alloys. Currently, there is ongoing research to analyze the different extraction methods of Pt, Pt alloys, and non-precious materials. As an environmentally friendly solution, researchers are trying to develop bio-synthesized nanoparticles as nanocatalysts for fuel cells and microbial fuel cells [113,114,115]. Table 2 represents several recent studies that have been carried out regarding bio-synthesized nanoparticles as catalysts for conventional fuel and microbial fuel cells.

### 2.2. Applications of Bio-Nanoparticles in Therapeutics

Bio-nanoparticles have garnered significant attention over the past decades owing to their excellent therapeutic capabilities. Their unique physicochemical properties, stability, solubility, and multi-functionality enhance their performance in various therapeutic applications, allowing for enhanced penetration and interaction with biological systems, targeted delivery, and efficacy. Moreover, their biocompatibility and ability to be functionalized for specific targeting further increase their effectiveness and safety in medical treatments [126]. In this section of the review, applications of bio nanoparticles in antioxidant, anticancer, anti-inflammatory, and antibacterial applications are discussed. 

Antioxidants are considered potent therapeutics for a variety of disease conditions. However, the use of these agents is doubtful in conventional therapy due to their instability, low permeability, and poor solubility [127]. Phytochemicals such as phenolic acids, terpenoids, and polyphenols from natural sources accompany substantial antioxidant potential. Bio-nanoparticles, functionalized with antioxidants derived from such bioactive compounds, have emerged as promising candidates for combating oxidative stress and are a heavily studied area in recent decades [128]. Cancer is considered to be an enormous challenge to human health. Bio-nanoparticle-based therapeutics have progressed significantly in the arena of cancer therapy, as conventional chemotherapy poses a multitude of limitations owing to the disadvantageous nature of the tumor microenvironment. Bio-nanoparticles offer a promising alternative to traditional chemotherapeutics with their enhanced capacities, including targeted delivery, selective anticancer effects, sustained release, and lower toxicity [129]. Various mechanisms have been proposed to explain the cytotoxicity mechanism of bio-nanoparticles, such as generation of reactive oxygen species (ROS), permeabilization of the mitochondrial outer membrane, activation of caspase-3, and specific DNA cleavage, all of which lead to apoptotic death of the cancer cell. There have been studies on bio-nanoparticles designed to treat cancer, including metallic nanoparticles from Ag, Au, Zn, and Cu, among the leading anticancer nanoparticles to date [130]. Inflammation is a localized physical response characterized by swelling, redness, pain, and other symptoms in the affected area in response to an infection or injury. Anti-inflammatory agents inhibit specific substances in the body that trigger inflammation [131]. Bio-nanoparticles are potent anti-inflammatory agents owing to their enhanced ability for selectivity and penetration and to restrict inflammatory messengers and enzymes compared to conventional therapy. Several bio-nanoparticles derived from metals and metal oxides, such as Ag, Au, Se, Cu, Ni, ZnO, FeO, and TiO_2_, are reported to be potent, with anti-inflammatory properties [132]. Multidrug-resistant bacterial pathogens are an escalating, highly debilitating threat worldwide, and conventional antibiotic therapeutics are rapidly becoming useless against the most resistant bacterial strains [133]. In pursuing alternative solutions, bio-nanoparticles have shown significant antibacterial activity, as they possess unique physical and chemical properties that enhance their interaction with microbial cells. The mechanisms through which bio-nanoparticles exhibit antibacterial effects include disruption of the bacterial cell membrane, generation of reactive oxygen species (ROS), and interference with cellular processes. The use of natural sources in the synthesis process imparts additional antibacterial properties due to the presence of bioactive compounds. Overall, the application of bio-nanoparticles in antibacterial treatments holds great promise for developing new, effective, and sustainable antimicrobial agents [134]. Table 3 provides examples of bio-nanoparticles synthesized from biological sources, including plants, fungi, bacteria, and algae, with their reported antioxidant, anticancer, anti-inflammatory, and antibacterial activities.

### 2.3. Applications of Bio-Nanoparticles in Waste Water Treatment

Due to the unique properties such as high surface area, reactivity, and functionality of bio-nanoparticles, they have emerged as highly effective agents in the wastewater treatment industry. Their properties lead to the removal of a wide range of contaminants, including heavy metals, organic pollutants, and pathogenic microorganisms. The wastewater or effluent containing non-biodegradable dyes and organic pollutants into the water reservoirs is mainly discharged from various industries, factories, and laboratories without any treatment, and it leads to a global environmental and health hazard [153]. Large quantities of dyes are used in many industrial applications such as textiles, papers, leathers, laser materials, laser printing, foodstuffs, cosmetics, xerography, gasoline, etc. And byproducts discarded from industries contain heavy metal ions and dyes, or both in most cases [154]. Furthermore, according to the estimated data, the total worldwide production of dyes is lost in their synthesis and dyeing process, which is over 15% [155]. The studies proved that most of these dyes are toxic and carcinogenic and reduce the light penetration of the aqueous systems. As a result, it causes serious concern to society due to the complex structures and non-biodegradable nature. This leads to negative effects on photosynthesis, is toxic for living organisms, is harmful to human health, and contributes significantly to the overall imbalance of the ecosystem [156].

Due to the high surface area and affinity for metal ions, carbon-based and metal-oxide nanoparticles have shown exceptional adsorption capacity on heavy metals like lead, mercury, and cadmium from wastewater [157,158]. Nanoparticles such as titanium dioxide show photocatalytic activity, and they are employed to break down organic contaminants, including pesticides, dyes, and pharmaceutical residues, converting them into less harmful substances. TiO_2_ and other metal oxides demonstrate high photocatalytic activity, but their effectiveness depends on several conditions, such as pH level, light intensity, and the presence of additional catalysts. pH levels, can affect the surface charge and light intensity directly impacts the electron-hole pairs which is a critical factor for photocatalysis. At the same time the availability of a co-catalysts can improve the overall efficiency [159]. Furthermore, silver and gold nanoparticles exhibit potential antimicrobial effects against harmful viruses and bacteria [160]. Moreover, the efficiency and sustainability of the wastewater treatment process are enhanced by magnetically responsive nanoparticles due to their easy recovery and reusable properties. Table 4 demonstrates a summary of recent research works carried out by scientists on the applications of bio-based nanomaterials in wastewater treatment. These advanced bio-nanomaterials provide a versatile and robust solution for addressing the difficult challenges of wastewater treatment, improving the efficiency, effectiveness, and sustainability while contributing to the protection of public health and the environment. The studies summarized in Table 4 are conducted as laboratory based research activities. Even though the results from these studies demonstrate promising outcomes, it should be noted that these research are conducted under controlled laboratory conditions. In industrial applications there may be number of additional challenges such as variations in environmental conditions, cost effectiveness and scalability.

### 2.4. Applications of Bio-Nanoparticles in the Energy Industry

The increasing demand for energy due to rapid technological advancement and global population growth has caused a formidable challenge for human existence [176]. Global power generation is moving towards greener generation methods, discouraging conventional methods such as coal power, fossil fuel, natural gas, etc., to overcome environmental challenges such as global warming [177,178]. Throughout the last few decades, researchers have been working on finding a successful alternative to fossil fuels for power generation. As a result, many promising biofuels have emerged, such as bioethanol, biogas, biohydrogen, biodiesel, algal biofuels [179,180], bio-methanol, etc. However, biofuels still must achieve many milestones in order to challenge the fossil fuel industry. With the recent development of nanotechnology, a great deal of research has been conducted to improve the production efficiency of biofuels and the performance of biofuels using nanotechnology [181,182,183]. Nanoparticles can improve the efficiency of the manufacturing process of biofuels, as they have higher reactive surfaces [184]. Today, scientists have taken one step further by introducing bio-nanotechnology, a combination of biology and nanotechnology, to the energy sector, which results in more environmentally friendly outcomes. At the same time, the health-related concerns to the human body from the applications of nanotechnology are comparatively reduced with bio-nanotechnology [185].

There are a number of different applications of bio-nanotechnology in the energy industry. When considering the most recent research trends, the green synthesis of nanoparticles from plants is rapidly increasing in popularity due to environmental friendliness and health concerns due to the utility of toxic chemicals. The bio-nanoparticles that various plants synthesize are used in numerous types of research to observe their performance as catalysts for the biofuel production process. In Table 5, a summary of the recent research related to the enhancement of biofuel production using bio-nano catalysts is presented. All the nanoparticles used were synthesized using different plant components, such as orange peels [185], pomegranate peels [186], *Euphorbia royleana* leaves [187], rice husk [188], and also animal wastes such as chicken-egg shell [189], etc. All the research has shown very positive results in improving the production efficiency of biofuels, which have a promising number of industrial applications for nanotechnology in the future energy sector.

## 3. Conclusions

Green synthesis of BNPs using plants, bacteria, fungi, and algae presents a promising and eco-friendly alternative to conventional methods. The diverse biochemical properties of these biological entities enable the production of nanoparticles with varied shapes and sizes, enhancing their applicability across multiple fields. BNPs have shown significant potential in therapeutics as antimicrobial, anti-inflammatory, antioxidant, and anticancer agents. Additionally, they are being integrated into fuel cells and energy generation systems, providing green energy solutions. In wastewater treatment, BNPs offer an effective and environmentally friendly approach to removing heavy metals, organic pollutants, and microbial contaminants. However, further research is essential to optimize synthesis processes, fully elucidate their mechanisms of action, and expand the scope of their applications. BNPs can address some of the pressing challenges in medicine, energy, and environmental sustainability, paving the way for innovative and sustainable technological advancements. The continued exploration and development of bio-nanoparticles for advancements in material engineering, hybridization strategies, and computational design hold great promise for the future, offering sustainable solutions that align with the growing demand for environmentally conscious technologies.

## Figures and Tables

**Table 1:** Green synthesis of NPs using biological materials.

Biological Material	Name	Morphology	Nanoparticle Size (nm)	Nanoparticle	Reference
**Plant**	*Abutilon indicum* leaves	Hexagonal	16	CuO	[33]
	*Aloe vera* leaves	Spherical	15–50	Ag	[34]
	*Bergenia ciliata* Rhizome	Spherical	20	CuO	[35]
	*Capparis spinosa* tissues	Spherical and semispherical	15–30	Ag	[36]
	*Catharanthus roseus* leaves	Hexagonal	35	ZnO	[37]
	*Coriandrum sativum* leaves	Spherical	15–50	Ag	[34]
	*Corymbia citriodora* leaves	Needle	21–28	Mn	[38]
	*Cuminum cyminum* seeds	Crystalline	15	TiO_2_	[39]
	*Cymbopogon citratus* leaves	Spherical	15–50	Ag	[34]
	*Cymbopogon olivieri*	Spherical	28	ZnO	[40]
	*Eucalyptus robusta* leaves	Spherical	16–23	Mn	[38]
	*Euphorbia helioscopia* leaves	Crystalline	30–100	Ag	[41]
	*Euphorbia pulcherrima* flowers	Cubical	16–54	CuO	[42]
	*Fragaria ananassa* fruits	Spherical	10–30	Cu	[43]
	*Hypericum perforatum* leaves	Spherical	20–50	MnO_2_	[44]
	*Lemna minor* tissues	Spherical	10–20	ZnO	[45]
	*Melia azedarach* leaves	Crystalline and spherical	50–71	TiO_2_	[46]
	*Mentha arvensis* leaves	Spherical	15–50	Ag	[34]
	*Nerium oleander* leaves	Spherical	26	Cu	[47]
	*Ocimum sanctum* leaf	Granular	-	CuO	[48]
	*Paullinia cupana* Kunth leaf extract	Spherical morphology	39–126	Ag	[49]
	*Phoenix dactylifera L* leaves	Cubic to spherical	12–97	Ag	[50]
	*Phyllanthus emblica* fruit	Large, irregularly shaped flakes	-	Cr_2_O_3_	[51]
	*Saccharum officinarum* stem	Spherical, square, cube, plate, rectangular	29–60	CuO	[52]
	*Triticum aestivum* seed	Spherical	21–42	CuO	[53]
**Bacteria**	*Aquaspirillum magnetotacticum*	Octahedral prism	40–50	Fe_2_O_3_	[54]
	*Arthrobacter gangotriensis*	Spherical	5–6	Ag	[55]
	*Arthrobacter kerguelensis*	Spherical	5	Ag	[55]
	*Bacillus cecembensis*	Spherical	7	Ag	[55]
	*Bacillus cereus*	Spherical	20–40	Ag	[56]
	*Bacillus indicus*	-	4–6	Ag	[55]
	*Bacillus megaterium D01*	Spherical	2.5	Au	[57]
	*Bacillus subtilis 168*	Hexagonal-octahedral	5–50	Au	[58]
	*Escherichia coli*	Wurtzite structure	2–5	CdS	[59]
	*Escherichia coli DH 5α*	Spherical	8–25	Au	[60]
	*Klebsiella aerogenes*	-	20–200	CdS	[61]
	*Lactobacillus casei*	Spherical	20–50	Ag	[62]
	*Magnetospirillum magnetotacticum*	Chain	47	Fe_3_O_4_	[63]
	*Plectonemaboryanum UTEX 485*	Cubic, octahedral	10–25	Au	[64]
	*Pseudomonas antarctica*	Spherical	11–12	Ag	[55]
	*Pseudomonas meridiana*	Spherical	5–6	Ag	[55]
	*Pseudomonas proteolytica*	Spherical	7	Ag	[55]
	*Rhodopseudomonas capsulate*	Spherical	10–20	Au	[65]
	*Serratia* sp. (ZTB29)	Polydisperse, spherical	20–40	CuO	[66]
	*Shewanella oneidensis*	-	1–5	UO_2_	[67]
	*Shewanella alga*	Triangular	10–20	Au	[68]
**Fungi**	*Alternata alternate*	Spherical	20–60	Ag	[69]
	*Aspergillus flavus*	-	1–8	Ag	[70]
	*Aspergillus flavus* TFR7	Spherical	12–15	TiO_2_	[71]
	*Aspergillus fumigates*	Spherical	5–25	Ag	[72]
	*Aspergillus niger*	Spherical	20	Ag	[73]
	*Aspergillus terreus*	Spherical	8	ZnO	[74]
	*Cariolus versicolor*	Spherical	25–75	Ag	[75]
	*Cladosporium cladosporioides*	Spherical	10–100	Ag	[76]
	*Fusarium oxysporum*	Spherical	8–14	Au-Ag alloy	[77]
	*Fusarium semitectum*	Crystalline spherical	10–60	Ag	[78]
	*Fusarium solani*	Spherical	5–35	Ag	[79]
	*Penicillium brecompactum*	Crystalline spherical	23–105	Ag	[80]
	*Penicillium fellutanum*	Spherical	5–25	Ag	[81]
	*Phanerochaete chrysosporium*	Pyramidal	50–200	Ag	[82]
	*Phoma glomerata*	Spherical	60–80	Ag	[83]
	*Rhizopus nigricans*	Round	35–40	Ag	[84]
	*Rhizopus stolonifer*	Spherical	25–30, 1–5	Ag Au	[85]
	*Saccharimyces cerevisae* broth	Spherical	4–15	Ag, Au	[86]
	*Trichoderma viride*	Spherical	5–40	Ag	[87]
	*Trichothecium* sp.	Spherical, rod-like, triangular	10–25	Au	[88]
	*Verticillium*	Spherical	21–25	Ag	[89]
	*Verticillium luteoalbum*	Triangular, hexagonal	10	Au	[90]
**Algae**	*Bifurcaria bifurcate*	Crystalline	5–45	CuO	[91]
	*Caulerpa racemosa*	Spherical and triangular	5–25	Ag	[92]
	*Chaetomorpha linum*	Nano-clusters	3–44	Ag	[93]
	*Chlamydomonas reinhardtii*	Round/rectangular	5–35	Ag	[94]
	*Chlorella vulgaris*	Crystalline	2–10	Au	[95]
	*Colpmenia sinusa*	Spherical	20	Ag	[96]
	*Cystophora moniliformis*	Spherical	50–100	Ag	[97]
	*Ecklonia cava*	Spherical and triangular	30	Au	[98]
	*Enteromorpha flexuosa*	Spherical	2–32	Ag	[99]
	*Enteromorpha flexuosa*	Spherical	2–32	Ag	[99]
	*Gracilaria gracilis*	Crystalline	25–50	ZnO	[100]
	*Jania rubins*	Spherical	12	Ag	[96]
	*Lemanea fluviatilis*	Spherical	5–15	Au	[101]
	*Padina gymnospora*	Spherical	53–67	Au	[102]
	*Prasiola crispa*	Spherical	5–25	Au	[103]
	*Pterocladia capillacae*	Spherical	7	Ag	[96]
	*Sargassum muticum*	Cubic	18	Fe_3_O_4_	[104]
	*Sargassum muticum*	Hexagonal wurtzite	30–57	ZnO	[105]
	*Sargassum muticum*	Spherical	5.4	Au	[106]
	*Tetraselmis kochinensis*	Spherical and triangular	5–35	Au	[107]
	*Ulva faciata*	Spherical	7	Ag	[96]

**Table 2:** Bio-synthesized nanoparticle applications in fuel-cells.

Biological Material	Synthesized NP	Characterization Technique	Nanoparticle Size and Morphology	Application	Method/ Measurement	Results	Ref.
*Escherichia coli* MC4100	*E. coli*-Pt/Pd (10%: 10%), E-coil-Pt (10%), and E-coil-Pd (10%) alloyed catalysts	Transmission electron microscope (TEM) X-ray diffraction (XRD)	5.2 nm	Fuel cell catalysts in polymer electrolyte fuel cell catalysts	The nanoparticles were synthesized by initially forming Pd nanoparticles on the *E. coli* cells, followed by Pt synthesis mediated by the Pd nanoparticles reducing Pt (IV) using K_2_PtCl_6_ and Na_2_PdCl_4_.	*E. coli*-Pt/Pd (10%:10%) showed better ECSA (electrochemical loaded area) compared to the other two samples.	[116]
*Escherichia coli* MC4100	Bio-Pd (desulfurized) nanoparticles Bio-Pd (E-coil) nanoparticles	TEM	30 nm	Fuel cell catalysts in proton exchange fuel cell catalysts	Four electrodes were manufactured: 1—Commercial Pt nanoparticles; 2—Commercial Pd nanoparticles; 3—Desulfurized Bio-Pd nanoparticles; 4—E-coil bio-nanoparticles.	Maximum power generated by each electrode was 0.13, 0.10, 0.11, and 0.04 watts.	[117]
Dairy wastewater	Cu-doped FeO	XRD Scanning electron microscope (SEM)	70–200 nm	Anode catalysts in a microbial fuel cell	Copper-doped iron oxide nanoparticles (Cu-doped FeO) were synthesized using phyto-compounds of the *A. blitum* plant.	161.5 W/m^2^ peak power density was delivered at 270 A/m^2^ current density.	[118]
*Citrobacter*	Bio-Pd nanoparticles	SEM XRD Energy-dispersive X-ray spectroscopy (EDS)	15.65–11.37 nm	Electrocatalysts for anion exchange membrane fuel cells	Bio-Pd was extracted from Pd (II) solution in the basal mineral medium using *Citrobacter*; 4 mg/cm^2^ and 2 mg/cm^2^ Bio-Pd nanoparticles were applied as anode catalysts.	4 mg/cm^2^ solution achieved 539.3 mW/cm^2^ maximum power density, which is 31.1% and 59.6% higher than that of 2 mg/cm^2^ solution and carbon rod.	[119]
Bean sprout	Bio-derived Co_2_P nanoparticles	SEM TEM X-ray photoelectron spectroscopy (XPS) XRD	10–100 nm	Electrocatalysts for anion exchange membrane fuel cells	Co_2_P nanoparticles were synthesized using the NH_3_ heat treatment.	Maximum power density of 172.2 mW/cm^2^ was achieved.	[120]
Pomegranate peel	Pd-NiO/C nanocatalyst	XPS XRD High-resolution scanning electron microscopy (HRSEM) SEM	5 nm	Pd support catalyst for alkaline direct ethanol fuel cell and CO_2_ electro-reduction	NiO nanoparticles were extracted from pomegranate, and Pd was added through the Pd (II) solution.	Cell output was reported as 117 mW.	[121]
Anaerobic digester sludge	Biosynthesized FeS nanoparticles	SEM XPS Field emission scanning Electron microscopy with energy dispersive X-Ray spectroscopy (FESEM-EDX) XRD	29.97 ± 7.1 nm	Anode of a microbial fuel cell	FeS was extracted from FeCl_3_ and Na_2_S_2_O_3_ using a biofilm	A maximum power density of 519 W/m^2^ was obtained	[122]
Banana, pineapple peels, and sugarcane bagasse	Biogenic platinum nanoparticles	UV–visible spectrophotometer Fourier-transform infrared spectroscopy (FTIR) XRD FESEM	Spherical shape 2–17 nm	For the improved methanol oxidation reaction in direct methanol fuel cell	Biosynthesis from banana peel, pineapple peel, and sugarcane bagasse.	ECSA values were reported for Pt extracted from sugarcane bagasse, banana peels, and pineapple peels as 94.58, 9.91, and 1.69 m^2^/g, respectively.	[123]
Jackfruit seed	Pt ornamented N-doped porous carbon	XPS TEM	5.12 nm	A catalyst for the oxygen reduction reaction	Carbon nanoparticles were derived from jackfruit seed.	ECSA of 68.5 m^2^/g and current density of 59.7 mA/cm^2^.	[124]
Butterfly wings	Bio-carbon substrate (porous carbon)	SEM TEM XRD	2.4–10 nm	A catalyst for the oxygen reduction reaction	Synthesized porous carbon from the black forewing of the butterfly *Troides aeacus* and synthesized Co_3_O_4_/CW.	Current density of 4.59 mA/cm^2^.	[125]

**Table 3:** Therapeutic applications of green synthesized bio-nanoparticles.

Biological Material	Synthesized NP	Characterization Technique	Characteristics of NP (Size and Morphology)	Application	Method/Measurement	Results	References
*Lactobacillus casei* 393 culture	Se	TEM SEM XPS EDX FTIR	50–80 nm Spherical	Antioxidant	H_2_O_2_-induced cell oxidative damage model and diquat-induced oxidative damage model	Inhibition of H_2_O_2_-induced oxidative damage and apoptosis and diquat-caused cytotoxicity in intestinal epithelial cells	[135]
Cell-free extracts of four strains of non-pathogenic *Enterococcus* sp.	Au	UV–vis FTIR TEM EDX	8–50 nm Spherical	Antioxidant	DPPH free radical scavenging assay	Significant antioxidant activity of 33.24–51.47%	[136]
*Aspergillus versicolor* ENT7	Ag	UV–vis FTIR TEM XRD	3–40 nm Spherical	Antioxidant	DPPH free radical scavenging assay	Antioxidant potential with IC50 value of 60.64 lg/mL	[137]
Marine endophytic fungi *Cladosporium cladosporioides*	Au	UV–vis FE-SEM XRD FTIR DLS EDX	30–60 nm Rough surface	Antioxidant	DPPH free radical scavenging assay, ferric reducing ability of plasma (FRAP) assay	Dose-dependent DPPH scavenging activity and moderate activity on FRAP-1.51 ± 0.03 mg of AAE/g sample	[138]
Red alga, *Lemanea fluviatilis* (L.)	Au	UV–vis XRD TEM FT-IR DLS	5–15 nm Nearly spherical, poly-dispersed, with the tendency to assemble together to form a chain-like structure	Antioxidant	DPPH free radical scavenging assay	Dose-dependent DPPH scavenging activity	[101]
Aqueous extract of aerial parts of *Alternanthera sessilis*	Ag	UV–vis TEM	10–30 nm Spherical	Anticancer	MTT assay against breast cancer MCF-7 cell line	Prominent anticancer activity, complete cell inhibition (99%) of MCF-7 cell line with 25 μg/mL, IC50 = 3.04 μg/mL	[139]
*Vitex negundo* L leaf extract	Ag	UV–vis FESEM TEM FTIR XRD EDX	5 to 47 nm Spherical and well dispersed	Anticancer	MTT assay against human colon HCT15 cancer cell line	High anticancer effects with IC_50_ of 20 μg/mL	[140]
*Mimosa pudica* leaf extract	Au	UV–vis FTIR XRD HR-TEM	12.5 nm Predominantly spherical and well dispersed	Anticancer	MTT assay against breast cancer cell lines (MDA-MB-231 and MCF-7)	Anticancer activity with IC_50_ of 4 µg/mL for MDA-MB-231 and IC_50_ of 6 µg/mL for MCF-7	[141]
Leaf extracts of *Olea europaea*	CuO	XRD FTIR SEM TEM	20–50 nm Spherical, smooth surfaces	Anticancer	MTT assay against AMJ-13 and SKOV-3 cancer cell lines	Cytotoxicity of IC_50_ for Brest cancer-AMJ-13—1.47 μg/mL and Ovarian cancer-SKOV-3—2.27 μg/mL	[142]
*Aspergillus niger* strain STA9	Cu	UV–vis FTIR DLS TEM SEM	5 to 100 nm Spherical, poly-dispersed	Anticancer	MTT assay against human hepatocellular carcinoma cell lines (Huh-7)	Significant cytotoxic effect against Huh-7 with IC_50_ 3.09 μg/mL value	[143]
Fruit extract of *Sambucus nigra*	Ag	UV–vis FTIR XRD TEM	20–80 nm Spherical	Anti-inflammatory	HaCaT cells exposed to UVB radiation, acute inflammation model	Significant anti-inflammatory activity with a decrease in cytokine production and reduction in edema formation	[144]
European cranberry bush (*Viburnum opulus)* fruit extract	Ag	UV–vis FTIR XRD TEM	10–50 nm Spherical	Anti-inflammatory	HaCaT cell line, exposed to UVB radiation, acute inflammation model	Significant anti-inflammatory activity with a decrease in cytokine production and reduction in edema formation	[145]
*Dalbergiaspinosa* leaf extract	Ag	UV–vis FTIR HR-TEM	18 8 ±4 nm Spherical	Anti-inflammatory	Human RBC membrane stabilization assay	Moderate anti-inflammatory effects with red blood cell membrane stabilization	[146]
*Prunus domestica* gum extract	Au	UV–vis FTIR SEM EDX	7–30 nm Spherical	Anti-inflammatory	Carrageenan-induced paw edema model	Significant anti-inflammatory effects by reducing paw edema	[147]
*Centratherum punctatum* Cass. leaf extract	Ag	UV–vis FTIR XRD SEM TEM XPS	50–100 nm Spherical	Anti-inflammatory	In vitro protein denaturation inhibition assay, human RBC membrane stabilization assay, and proteinase inhibitory assay	Significant anti-inflammatory effects via protein denaturation inhibition, RBC membrane stabilization, and proteinase inhibition	[148]
Callus extract of *Cinnamonum camphora*	Ag	UV–vis TEM SEM-EDX DLS FT-IR XRD	5.47–9.48 nm Spherical, homogenous distribution	Antibacterial	Minimum inhibitory effect (MIC) via well diffusion method against *E. coli*, *P. aeruginosa*, *S. aureus*, and *B. subtilis*	MIC = 10 µg/mL for *S*. *aureus* and *B. subtilis*; MIC = 20 µg/mL for *E. coli* and *P.* *aeruginosa*	[149]
*Aspergillus niger* strain STA9	Cu	UV–vis FTIR SEM TEM DLS	5 to 100 nm Spherical, poly-distributed	Antibacterial	In vitro agar well diffusion assay against *E. coli*, *S. aureus*, *K. pneumoniae*, *Micrococcus luteus*, and *B. subtilis*.	Inhibition zone of 19, 21, 16, 20, and 17 mm against *E. coli*, *S. aureus*, *K. pneumoniae*, *Micrococcus luteus*, and *B. subtilis*, respectively	[143]
*Bacillus subtilis* culture	Ag	UV–vis TEM FT-IR	3–20 nm Spherical or roughly spherical	Antibacterial	Minimum inhibitory effect (MIC) via agar disc diffusion assay against MRSA, *S. epidermidis*, *K. pneumoniae*, *E. coli*, and *C. albicans*	Significant antimicrobial efficacy; MIC of 230, 180, 200, 100, and 0.300 mgmL^−1^ for MRSA, *S. epidermidis*, *E. coli*, *C. albicans*, and *K. pneumonia*, respectively.	[150]
*Psidium guajava* leaf extract	FeO	XRD SEM HR-TEM UV–vis	1–6 nm Morphology: ND	Antibacterial	Minimum inhibitory effect (MIC) via well diffusion method against *S. aureus*, *E. coli*, *P. aeruginosa*, *Shigella*, *S. typhi*, and *Pasteurella*	Strong antibacterial activity chiefly against *E. coli* and *S. aureus* at low concentration	[134]
Ethanolic extract from *Moringa oleifera* seed residue	Ag	SEM XRD DLS	90–180 nm Spherical	Antibacterial	Growth inhibition of *E. coli* BL21(DE3)	Significant inhibition of bacterial growth, elongating the lag phase in a dose-dependent manner	[151]
*Tetraclinis articulata* leaf extract	Ag	UV–vis SEM FTIR	Spherical 80 nm	**Anti-inflammatory** **Antioxidant** **Cytotoxicity**	Cell proliferation tests	Significant anti-inflammatory and antioxidant capacity, with an activity level similar to the control but without causing harm to cells	[152]

**Table 4:** Bio-synthesized nanoparticle applications in wastewater treatment.

Biological Material	Synthesized NP	Characterization Technique	Characteristics of NP (Size and Morphology)	Application	Method/Measurement	Results	Ref.
*Citrus aurantifolia* (keylime)	CuO	XRD UV–vis SEM FTIR	Size of ~22 nm and 3.48– 3.51 eV band gap	Degradation of organic pollutants	Photocatalytic activity antibacterial activity	91% dye removal; exhibited good antibacterial activity	[161]
*Cupressus sempervirens* (Mediterranean cypress)	CuFe_2_O_4_	XPS AFM SEM TEM	Nanosheet thickness ∼2.5 nm Size 20–30 nm	Degradation of organic pollutants	Catalytic activity measurements	Observed greater catalytic performances, reusability, and recovery	[162]
*Nerium oleander*	CuO	FTIR SEM EDX XRD	Size 21 nm	Degradation of organic pollutants	Adsorbent measurements	Effective and eco-friendly nano-adsorbent treatment ability shown for the colored water	[163]
Sal seed de-oiled cake	CuO	UV–vis		Degradation of organic pollutants	Adsorbent measurements	Removed three azo dyes, namely Erichrome black T (EBT), Congo red (CR), and reactive violet 1 (RV1). Performed 80% dye removal efficiency, with re-usability	[164]
*Portulaca oleracea*	CuO	UV–vis FTIR XRD TEM EDX DLS Zeta potential	Spherical and crystalline Size 5–30 nm Surface plasmon resonance 275 nm	Degradation of organic pollutants	Antimicrobial activity and tanning wastewater treatment	The catalytic activity of nanoparticles in darkness recorded 70.3% decolorization, while sunlight irradiation improved the catalytic activity of nanoparticles to 88.6%; reduced the heavy metal percentage in wastewater	[165]
Brassica leaf	CuO	EDX FTIR SEM XRD UV–vis TEM EDAX	Size 50 nm	Degradation of organic pollutants	Adsorbent measurements Determination of pH (point of zero charge)	The percentage of dye adsorbent increased up to 99%; the dye removal efficiency decreased with increasing the amaranth dye concentration, with point of zero charge at pH 7.7	[166]
*Ruellia tuberosa*	ZnO	UV–vis FTIR TEM EDAX	Rod-shaped nanoparticles Size 40–50 nm	Degradation of organic pollutants	Photocatalytic property Degradation of synthetic dyes	Maximum dye removal percentages were 94% for methylene blue and 92% for malachite green	[167]
*Phoenix dactylifera* waste	ZnO	UV–vis EDX XPS FTIR XRD	Spherical shape nanoparticles Size 30 nm	Degradation of organic pollutants Disinfection	Dye degradation and antibacterial performance (disc-diffusion method)	Degradation efficiency was 90% for methylene blue and eosin yellow dyes; demonstrated significant antibacterial effects on Gram-positive and Gram-negative bacterial strains	[168]
*Eucalyptus* spp. Fresh, green leaves	ZnO	FESEM XRD BET TGA HRTEM EDX FTIR	Irregular in shape Size 40 nm Nanoparticles contained 76.6% zinc and 23.3% oxygen	Degradation of organic pollutants	Dye adsorption measurements (Langmuir and Temkin isotherm models) pH measurements	Maximum adsorption capacities were 48.3 mg/g for Congo red dye and 169.5 mg/g for malachite green dye; maximum removal was achieved at pH 6.0 and pH 8.0 for Congo red and malachite green dyes, respectively.	[169]
*Persea americana* (Avocado) oil	Au	UV–vis TEM FTIR DLS XRD	Spherical, decahedron, and triangular 48.8 ± 24.8 nm	Degradation of organic pollutants Removal of heavy metals	Antioxidant activity Dye adsorption measurements Photocatalytic activity	Enhanced antioxidant 30%, 40 μL photocatalytic decomposition of the methylene blue > 84%, 10 mg/L, 0.0057664 min	[170]
*Alpinia nigra* leaf	Au	UV–vis FTIR XRD TEM	Spherical 21.52 nm	Degradation of organic pollutants; Disinfection	Antioxidant activity Antimicrobial activity Photocatalytic activity	Antioxidant activity with IC50 value of 52.16 µg/mL; resistance to the growth of both Gram-positive and Gram-negative bacteria	[171]
*Allium cepa*	Ag	SEM TEM XRD ATR-FTIR	Spherical 50–100 nm	Degradation of organic pollutants	Photocatalytic activity Antimicrobial activity	Photocatalytic decomposition of the methylene blue > 80%	[172]
*Cynara cardunculus* Leaf	Fe_3_O_4_	UV–vis SEM XRD	Semi-spherical, aggregated 13.5 nm	Degradation of organic pollutants (kinetic adsorption model)	Photocatalytic activity	Photocatalytic decomposition of the methylene blue > 90%	[173]
*Plantago major* leaf	FeO	UV–vis TEM XRD FTIR	Spherical 4.6–30.6 nm	Degradation of organic pollutants	Photocatalytic activity	Methyl orange dye removal efficiency of 83.33% after a 6 h process	[174]
*Moringa oleifera* leaf	ZnO NP	UV–vis XRD FE-SEM TEM	Spherical 14 nm	Effectively breaking down the organic compounds present in synthetic petroleum wastewater	Photocatalytic activity	Degradation efficiency of green-ZnO, which, within 180 min of irradiation, achieved removal rates of 51%, 52%, 88%, and 93% for phenol and O-Cresol	[175]

**Table 5:** Recent studies on the utilization of bio nano-catalysts for biofuel production.

Biological Material	Synthesized NP	Characterization Technique	Characters of NP (Size and Morphology)	Application	Method/Measurement	Results	Ref.
Orange peel	Carbon quantum dots	DLS XRD TEM FTIR	-	Bio-nano emulsion fuel; Fuel was prepared with diesel, biodiesel, nanoparticles, and distilled water. The study was performed to observe the performance and emission of the bio-nano emulsion fuel using a four-stroke engine.	Fuel samples were prepared using three steps. Water was used as an intermediate fuel, as carbon quantum dots are highly stable in water. Fatty acids and neutral salt were used to stabilize water in diesel. Engine power, fuel consumption, and torque were measured.	The optimum concentration ratio of water 5 vol%/nanoparticle 60 ppm resulted in a 21% power increase at 2700 rpm	[185]
Pomegranate peel	Magnetic Fe_2_O_3_	XRD DLS Zeta potential analysis SEM EDX	28–80 nm Hexagonal/round-shaped	Biodiesel production; the study was performed to produce biodiesel from hazardous algae in water using bio-synthesized magnetic nanomaterials.	The optimum microalgae harvest conditions were determined using RSM (response surface methodology). Experimental data were obtained for the amount of γ-Fe_2_O_3_, stirring speed, mixing time, and temperature.	The optimal microalgae harvest conditions were identified as 56 mg L^−1^, 310 rpm, 48 s, and 22.5 °C, respectively. The biodiesel produced satisfied the ASTM D6751 standard, the specification for biodiesel fuel, excluding acid levels.	[186]
Chicken-egg shell	Calcium oxide (CaO)	FTIR TEM XRD SEM BET	75 nm Heterogeneous	Biodiesel production; the study was performed to produce biodiesel from microalgae dry biomass using bio-calcium oxide (CaO) as a nanocatalyst.	The transesterification process was used to produce biodiesel with chicken-egg shell waste-synthesized calcium oxide (CaO) nanocatalysts. Reaction parameters such as catalyst ratio, reaction time, and interactions with stirring rate were studied with RSM (response surface methodology).	The 1.7% (*w*/*w*) nanocatalysts ratio provided the optimum reaction performance with 86.41% biodiesel yield.	[189]
*Euphorbia royleana* plant	Bi_2_O_3_ (bismuth oxide)	XRD SEM EDX FTIR	-	Biodiesel production; the study was performed to produce biodiesel from the *Cannabis sativa* plant, and bio-synthesized Bi_2_O_3_ (bismuth oxide) nanoparticles were used as a nanocatalysts.	The seed oil of *Cannabis sativa* was used as the biomass for the synthesis of biodiesel. Reaction parameters of the transesterification reaction, such as catalyst concentration, reaction time, molar ratio, and temperature, were analyzed.	The 1.5 *w*/*w*% Bi_2_O_3_ (bismuth oxide) catalyst sample provided the optimum reaction conditions with 92% methyl ester yield at 12:1 methanol/oil, 92 °C, 210 min reaction duration.	[187]
Rice husk	Nano-bifunctional super magnetic RHC/K_2_O/Fe catalysts	XRD FTIR BET TGA VSM	-	Biodiesel production; the study was performed to study the effect of RHC/K_2_O/Fe catalyst for the transesterification of used cooking oil to produce biodiesel.	The reaction parameters such as temperature, reaction duration, methanol/oil molar ratio, and catalyst concentration were analyzed.	The RHC/K2O-20%/Fe-5% catalyst 4 wt% sample provided the optimum reaction conditions with a yield of 98.6% at 75 °C, 4 h reaction time, and methanol/oil 12:1.	[188]
*Madhuca indica* oil	Reusable magnetic multimetal nano-catalyst (Fe_3_O_4_·Cs_2_O)	XRD FTIR FE-SEM		Used for esterification and transesterification of *Madhuca indica* oil to produce biodiesel.	Variables involved in the process include catalyst concentration, the molar ratio of methanol to oil, reaction temperature, and duration of the reaction.	A peak conversion of 97.4% was achieved under the specified conditions of an 18:1 methanol-to-oil ratio, 7 wt% catalyst loading, a reaction temperature of 65 °C, and a reaction duration of 300 min.	[190]

## Data Availability

No data were used for the research described in the article.

## Abbreviations

The following abbreviations are used in this manuscript:

ATR-FTIR	Attenuated total reflectance Fourier-transform infrared spectroscopy
BET	Brunauer–Emmett–Teller
DLS	Dynamic light scattering
EDAX	Energy-dispersive X-ray spectroscopy
EDS	Energy-dispersive X-ray spectroscopy
FESEM	Field emission scanning electron microscopy
FESEM-EDX	Field emission scanning electron microscopy with energy dispersive X-ray spectroscopy
FTIR	Fourier-transform infrared spectroscopy
HRSEM	High-resolution scanning electron microscopy
HRTEM	High-resolution transmission electron microscopy
SEM	Scanning electron microscopy
SEM-EDX	Scanning electron microscopy with energy dispersive X-ray spectroscopy
TEM	Transmission electron microscopy
TGA	Thermogravimetric analyzer
UV–vis	Ultraviolet–visible spectrophotometer
